# Metagenomic analysis of planktonic microbial consortia from a non-tidal urban-impacted segment of James River

**DOI:** 10.1186/s40793-015-0062-5

**Published:** 2015-09-19

**Authors:** Bonnie L. Brown, Rebecca V. LePrell, Rima B. Franklin, Maria C. Rivera, Francine M. Cabral, Hugh L. Eaves, Vicki Gardiakos, Kevin P. Keegan, Timothy L. King

**Affiliations:** Department of Biology, Virginia Commonwealth University, 1000 W Cary Street, Richmond, VA 23284 USA; Environmental Epidemiology Division, Virginia Department of Health, 109 Governor Street, Richmond, VA 23219 USA; Department of Microbiology and Immunology, Virginia Commonwealth University, 1101 East Marshall Street, Richmond, VA 23298 USA; School of Life Sciences, Virginia Commonwealth University, 1000 W Cary Street, Richmond, VA 23284 USA; Virginia Department of Conservation and Recreation, Soil and Water Conservation, 600 East Main Street, Richmond, VA 23219 USA; Argonne National Laboratory, Biosciences Division, 9700 South Cass Avenue, Argonne, IL 60439 USA; US Geological Survey, Aquatic Ecology Branch, Leetown Science Center, 11649 Leetown Road, Kearneysville, WV 25430 USA

**Keywords:** James River, Virginia, Temperate urban river ecosystem, Industry, Pathogen, Water-borne disease

## Abstract

Knowledge of the diversity and ecological function of the microbial consortia of James River in Virginia, USA, is essential to developing a more complete understanding of the ecology of this model river system. Metagenomic analysis of James River's planktonic microbial community was performed for the first time using an unamplified genomic library and a 16S rDNA amplicon library prepared and sequenced by Ion PGM and MiSeq, respectively. From the 0.46-Gb WGS library (GenBank:SRR1146621; MG-RAST:4532156.3), 4 × 10^6^ reads revealed >3 × 10^6^ genes, 240 families of prokaryotes, and 155 families of eukaryotes. From the 0.68-Gb 16S library (GenBank:SRR2124995; MG-RAST:4631271.3; EMB:2184), 4 × 10^6^ reads revealed 259 families of eubacteria. Results of the WGS and 16S analyses were highly consistent and indicated that more than half of the bacterial sequences were *Proteobacteria*, predominantly *Comamonadaceae*. The most numerous genera in this group were *Acidovorax* (including iron oxidizers, nitrotolulene degraders, and plant pathogens), which accounted for 10 % of assigned bacterial reads. *Polaromonas* were another 6 % of all bacterial reads, with many assignments to groups capable of degrading polycyclic aromatic hydrocarbons. *Albidiferax* (iron reducers) and *Variovorax* (biodegraders of a variety of natural biogenic compounds as well as anthropogenic contaminants such as polycyclic aromatic hydrocarbons and endocrine disruptors) each accounted for an additional 3 % of bacterial reads. Comparison of these data to other publically-available aquatic metagenomes revealed that this stretch of James River is highly similar to the upper Mississippi River, and that these river systems are more similar to aquaculture and sludge ecosystems than they are to lakes or to a pristine section of the upper Amazon River. Taken together, these analyses exposed previously unknown aspects of microbial biodiversity, documented the ecological responses of microbes to urban effects, and revealed the noteworthy presence of 22 human-pathogenic bacterial genera (e.g., *Enterobacteriaceae*, pathogenic *Pseudomonadaceae*, and ‘*Vibrionales'*) and 6 pathogenic eukaryotic genera (e.g., Trypanosomatidae and Vahlkampfiidae). This information about pathogen diversity may be used to promote human epidemiological studies, enhance existing water quality monitoring efforts, and increase awareness of the possible health risks associated with recreational use of James River.

## Introduction

James River is an historical, cultural, and economic icon in North America [1] and one of the largest tributaries of the Chesapeake Bay. The James River ecosystem once provided provisioning services and transport for First Americans and European colonists, and the river has more recently been characterized as the economic engine of Virginia because it supports multiple economic services such as industry, commerce, and recreation. The James River watershed is home to more than 25 million Virginians, and its land use is: 71 % forested, 16 % agricultural, 5 % urban, and 8 % other [2]. In the watershed, there are >1500 point sources permitted to discharge pollutants from municipal and industrial outfalls, CSOs, and aging/failing sewage treatment facilities (data obtained from Virginia Department of Environmental Quality via Freedom of Information Act). The river also receives nonpoint source pollutants that derive from urban, agricultural, wildlife, and transportation runoff. Contaminants include sediment, nutrients (especially nitrogen and phosphorus), PBTs, and non-PBTs [3], as well as pathogens capable of causing illnesses and WBDOs. In fact, in the United States, WBDOs are increasing exponentially [4], and the potential for disease transmission is especially high in the James because its beaches and waters are heavily accessed for recreation (swimming, kayaking, river-boarding) and education (especially summer camps for children). Though there are methods available to assess the abundance of some of the more common disease-causing agents, each pathogen must be examined separately, and there are few methods available that consider the risk of multiple pathogens simultaneously. High-throughput sequencing is a cultivation-independent method that provides information about epidemiologically-relevant organisms that could enhance efforts to prevent, control, and better predict WBDOs thereby improving public health [5]. Current recreational water monitoring practices (e.g., *E coli* and coliform testing) serve only as coarse indictors of potential contamination, and provide little information on the diversity, source, ecology, or evolution of organisms that cause WBDOs. Metagenomic methods used in the nascent field of public health genomics could help address such questions, but studies thus far have focused narrowly on oral, nasal, gastric, and vaginal microbiota and their role in human health. On a few occasions, metagenomic techniques have been used to detect the occurrence of specific pathogens in the environment, such as coliforms, *Mycobacterium tuberculosis*, *Salmonella enterica* subsp. *enterica*, and *Vibrio cholerae* [6–9]; however, such approaches are tedious, expensive, or simply impractical for use in routine monitoring programs, and thus this sort of assessment has not found wide application at the larger ecogenomic scale. This report is the first installment of the James River Metagenome Project.

## Site information

The segment of James River near downtown Richmond, Virginia (USA) is non-tidal within the fall zone (Piedmont Upland transitioning to the Atlantic Coastal Plain, Table 1). This site has both recreational and monitoring relevance. This location occurs in a highly-urbanized area, where storm water runoff carries pollutants such as oil, sediment, chemicals, heavy metals, pet waste, and lawn fertilizers directly to the river. James River traverses more than 700 km^2^ of impervious surface between Lynchburg and Richmond. Along this distance, construction sites, power plants, failing sewer systems, and industrial activities contribute substantial amounts of contaminants. Further, this sampling location is impacted by activities in the entire watershed upstream, especially the large cities of Charlottesville and Lynchburg. For example, between Richmond and Lynchburg, there are 170 active industrial discharge sites and 92 sources permitted to discharge directly into James River without pre-treatment. The sampled portion of James River is proximal to numerous highly trafficked bridges and downstream of a large urban park with abundant riparian and aquatic wildlife (e.g., turtles, ducks, geese, heron, amphibians, and reptiles). The city of Richmond has one of the largest CSO systems on the East Coast, and our sampling station is affected by discharge from 19 CSOs within 10 km . This segment of the river has been included in the state’s Impaired Waters List for fecal coliforms for over a decade and, although the government regulates only certain bacterial TMDLs, there is sufficient evidence to assume that the water is impaired with regard to other pollutants according to the Clean Water Act [10]. The Virginia Department of Health also has a long-standing fish consumption advisory for this section of the river due to elevated levels of PCBs.

**Table 1:** Study information

Label	JREM1 Riffles_WGS	JREM1 Riffles_16S
MG-RAST ID	4532156.3	4631271.3
SRA ID	SRR1146621	SRR2124995
Study Name	James River Epidemiological Metagenome	James River Epidemiological Metagenome
NCBI BioProject	PRJNA236764	PRJNA236764
Relevance	Ecology, Epidemiology	Ecology, Epidemiology

## Metagenome sequencing information

### Metagenome project history

We characterized a metagenome from the non-tidal James River near Richmond, Virginia. This stand-alone river study was conceived to investigate the potential of environmental metagenomic analysis in public health, and began with a sample collected in September 2012, the analysis of which is presented in this report. We used MG-RAST [11], MEGAN [12], and RDP [13] to categorize the sequence data and to identify taxa that contribute to the ecology of this river ecosystem so as to better understand how the microbial consortia respond to urbanization, pollution, and other anthropogenic influences. The data are accessed in NCBI and MG-RAST (Table 1).

## Sample information

James River sampling took place on 21 September 2012 during an historically typical late summer month when neither drought nor excessive precipitation events occurred within the two weeks preceding sample collection. This minimized potential effects of severe weather and CSO inputs. At the time of collection, the physicochemical parameters of the water column were: 21.9 °C, 79 mg L^−1^ dissolved oxygen, 8.4 pH, 86 m^3^ s^−1^ discharge, 9.7 FNU turbidity, 250 CFU 100 mL^−1^ fecal coliform, and 175 CFU 100 mL^−1^*E. coli*. These parameters, although not pristine, indicate that the water was unimpaired at the time of sample collection according to the Clean Water Act [10] and state water quality standards.

## Sample preparation

Water (20 L) was collected by wading to mid-stream, waiting until disturbed sediment had dissipated, and then inserting a clean collection vessel to mid-water (0.5 m below the water surface), tipping to collect, and capping underwater. The water was held at ambient temperature during transport to the laboratory (~10 min) for immediate processing (Table 2). After mixing, a 3-L subsample was gently filtered through 0.2-μm Sterivex™ filters (Millipore, Billerica, MA) using a combination of gravity and vacuum (200–300 mm Hg). It is possible that this pressure may have disrupted some soft-bodied protists, limiting our ability to detect this group. Free viruses and some eDNA also are likely to have passed through the 0.2-μm filter.

**Table 2:** Sample information

Label	Mayo South_WGS	Mayo South_16S
Biome	0000088 Large river	0000088 Large river
Feature	00000012 Hydrographic	00000012 Hydrographic
Material	449393 Fresh water	449393 Fresh water
Latitude and Longitude	37.527941 N, −77.436144 W	37.527941 N, −77.436144 W
Vertical distance	0.5 m	0.5 m
Geographic Location	James River, Virginia	James River, Virginia
Collection date and time	21 Sep 2012; 14:00 h (EST)	21 Sep 2012; 14:00 h (EST)

## DNA extraction

DNA was isolated using the Sterivex™ PowerWater™ DNA extraction (MO BIO, Carlsbad, CA) within 2 h of collection according to the manufacturer’s instructions, a procedure that included enzymes, heat, and bead beating to ensure nucleic acid release from endospore-forming and Gram negative bacteria. Nucleic acid quality was checked via Experion™ DNA 12 K Analysis kit.

## Library generation

Nucleic acid quantity was verified using the Quant-iT DNA kit (Life Technologies, Grand Island, NY), and adjusted to 50 ng μL^−1^ prior to WGS library preparation using the Ion Plus Fragment Library kit (Life Technologies, Grand Island, NY). To alternatively assess taxonomic diversity of the microbial community, a library was made that targeted the bacterial 16S rRNA gene; four replicate fusion PCR libraries targeting 16S [14] were performed using the same DNA sample as the shotgun metagenome. The amplicons were quantified using Bioanalyzer and pooled in equimolar amounts prior to sequencing.

## Sequencing technology

Sequencing of the WGS library was accomplished using the Ion Torrent PGM semiconductor sequencing platform (Life Technologies, Grand Island, NY), the Ion PGM™ 200 Sequencing Kit, and one 318 chip. The run generated 0.61Gb data (Table 3). The 16S targeted library generated 1.15Gb data from one 1 × 300 bp lane on MiSeq (Illumina, San Diego, CA).

## Sequence processing

Quality control for the WGS run was performed on the MG-RAST server, and filtering for 16S amplicons was accomplished in BaseSpace (quality scores ≥30). After quality control filtering, the James River WGS metagenome consisted of 3.4 × 10^6^ reads with an average length of 133 ± 43 bp (Table 2) and the 16S rDNA amplicon library consisted of 3.9 × 10^6^ reads with an average length of 292 ± 0 bp (Table 4).

**Table 3:** Library information

Label	Riffles_WGS	Riffles_16S
Sample label	Mayo South	Mayo South
Sample preparation method	Mobio Sterivex™ PowerWater™ DNA Kit	Mobio Sterivex™ PowerWater™ DNA Kit*
Library preparation	Ion Plus Fragment Library kit	16S fusion PCR [14]
Sequencing platform(s)	Ion Torrent PGM	Illumina MiSeq
Sequencing chemistry	PGM Sequencing 200 Kit (318 chip)	MiSeq Reagent Kit Version 3 (600 cycles PE)
Sequence size (GBp)	0.61	1.15
Number of reads	4.07 × 10^6^	4.11 × 10^6^
Mate pairs or paired ends	NA	empty
Sequencing library insert size	150–250	300
Average read length	133	292
Standard deviation for read length	43	0

* DNA template from the same 3 L water sample was used for both the WGS and 16S libraries

**Table 4:** Sequence processing

Label	Riffles_WGS	Riffles_16S
Tool(s) used for quality control	MG-RAST	BaseSpace
Number of sequences removed by QC	0.61 × 10^6^	0.18 × 10^6^
Number of sequences that passed QC	3.45 × 10^6^	3.93 × 10^6^
Number of artificial duplicate reads	0.13 × 10^6^	NA

## Metagenome processing

No assembly was performed for either data set (Table 5).

**Table 5:** Metagenome statistics

Label	JREM1 Riffles_WGS	JREM1 Riffles_16S
Libraries used	Riffles_WGS	Riffles_16S
Assembly tool(s) used	NA	NA
Number of contigs after assembly	NA	NA
Number of singletons after assembly	NA	NA
minimal contig length	NA	NA
Total bases assembled	NA	NA
Contig n50	NA	NA
% of sequences assembled	NA	NA
Measure for % assembled	NA	NA

## Metagenome annotation

Shotgun sequence data were analyzed using bioinformatic tools on the MG-RAST server to predict rDNA, gene, and protein functions. The MG-RAST analysis was performed using the BLAT annotation algorithm [15] against the M5NR protein Db using default parameters. Targeted 16S rDNA amplicon sequences of at least 100 bp were analyzed using the Illumina 16S Metagenomics App (v1.0.0) for taxonomic classification using an Illumina-curated version of the May 2013 GreenGenes taxonomic Db and default settings (Table 6).

**Table 6:** Annotation parameters

Label	JREM1 Riffles_WGS	JREM1 Riffles_16S
Annotation system	MG-RAST	BaseSpace 16S Metagenomics App (V1.0.0)
Gene calling program	Default (FragGeneScan)	Illumina implementation of RDP
Annotation algorithm	Default (BLAT)	NA
Database(s) used	M5NR, SEED, KEGG	GreenGenes

## Postprocessing

Whole-genome shotgun sequence data were compiled and assessed using three methods (MG-RAST, MEGAN, and crAss [16]). Bar charts of normalized counts of the highest representative taxa were constructed using the MG-RAST output with an e-value cutoff of 1e-5, 60 % identity, and a minimum alignment length of 30. Comparative metagenomic similarity was quantified between the James River and 17 other putatively similar, publicly available MG-RAST metagenomic read sets (4532156.3, 4440411.3, 4440413.3, 4440423.3, 4441132.3, 4441590.3, 4442450.3, 4467029.3, 4467420.3, 4467059.3, 4494863.3, 4516288.3, 4534334.3, 4534338.3) using principal coordinates analysis (M5NR Db, e-value cutoff −5, 60 % identity, data normalized using the MG-RAST default normalization procedure, minimum alignment length of 15 bp). Functional aspects of the James River WGS metagenome were compared with 13 other aquatic metagenomes on MG-RAST (2 large river samples, 4 lakes, 3 aquaculture, 2 sludge, and 2 Chesapeake Bay) using principal coordinates analysis (Subsystems Level 1, e-value cutoff 1e-5, 60 % identity, data normalized using the default normalization procedure and a minimum alignment length of 15 amino acids) and KeggMapper (e-value cutoff 1e-5, 60 % identity, minimum alignment length of 15 amino acids). Prior to a local BlastN, WGS reads <50 bp were removed and quality trimmed to ≥ Phred 20 using Genomics Workbench (CLCbio, Cambridge, MA). BlastN was performed locally using the quality- and size-filtered shotgun genomic data against the NCBI-nt reference Db using the default conditions of megablast. The resulting Blast report was then parsed and analyzed using MEGAN (ver 4.70.4) yielding a taxonomic “species profile.” To compare genetic similarity of the James River WGS metagenome with other aquatic WGS metagenomes, including those that were not available through MG-RAST (SRA001012, SRR091234, SRR063691), the algorithm crAss [16] was used to estimate genetic distances based on the characteristics of “cross-contigs” obtained by cross-assembly of all sets of reads using Genomics Workbench with the following parameters: mismatch 3, insertion 3, deletion 3, length fraction of 50 %, and similarity fraction of 90 %.

## Metagenome properties

Unlike some other WGS metagenomes that exhibit bimodal GC distribution [17–20], this James River metagenome exhibited a unimodal peak (WGS: 49 ± 9 % and 16S: 51 ± 2 %), well within the range observed and suggested as a freshwater hallmark (46–65 % [18]). The targeted 16S metagenome provided roughly the same number of reads as the WGS analysis and an order of magnitude lower numbers of CDSs and functional assignments (Table 7).

**Table 7:** Metagenome properties

Label	JREM1 Riffles_WGS	JREM1 Riffles_16S
Number of contigs	NA	NA
GBp	0.61	1.15
Number of features identified	3.13 × 10^6^	3.53 × 10^6^
CDS	2.16 × 10^6^	2.47 × 10^5^
rRNA	436,985	56,138
Functional Categories	914,799	7,299
CDSs with COG	1.50 × 10^6^	1,091
CDS with SEED subsystem	2.50 × 10^6^	146,907
Alpha diversity	316	20

## Taxonomic diversity

Reads resulting from James River WGS were overwhelmingly assigned to the *Bacteria* domain (97.5 % by MG-RAST, 97.7 % by Blast), *Eukaryota* accounted for 2 % of assignments (MG-RAST and Blast), and the remaining assignable reads were *Archaea* (0.3 % by MG-RAST, 0.1 % by Blast) and virus or plasmid (0.2 % by MG-RAST, 0.07 % by Blast). Reads resulting from the 16S library were bacterial (95.5 %) and viral (4.5 %, a sequencing control contaminant [21]). Taxonomy based on predicted proteins and rRNA genes (MG-RAST) generally mirrored the major taxa predicted by BlastN (MEGAN). The taxonomic profiles of the major bacterial groups based on WGS reads and 16S reads (Table 8) were largely concurrent, consistent with other research where WGS and 16S data were compared [17, 22]. The major differences between the WGS and 16S rDNA amplicon-based taxonomic profiles assigned to Class were *Cytophagia* (7.3 % in the WGS library and not detected in the 16S library), *Chlorobia* (0.2 % in the WGS library and not detected in the 16S library), and *Synergistia* (0 % of WGS and 0.5 % of 16S reads).

**Table 8:** Taxonomic composition of the sample

Domain	Phylum	Class*	JREM1 Riffles_WGS	JREM1 Riffles_16S
Archaea			9138	
	*Crenarchaeota*	*Thermoprotei*	1246	
	*Euryarchaeota*	*Archaeoglobi*	291	
	*Euryarchaeota*	*Halobacteria*	1799	
	*Euryarchaeota*	*Methanococci*	806	
	*Euryarchaeota*	*Methanobacteria*	692	
	*Euryarchaeota*	‘*Methanomicrobia’*	2425	
	*Euryarchaeota*	*Thermococci*	691	
	*Euryarchaeota*	*Thermoplasmata*	271	
	*Euryarchaeota*	unclassified	377	
	*Thaumarchaeota*	unclassified	270	
	unclassified	unclassified	115	
Bacteria			3420971	3704883
	*Acidobacteria*	*Acidobacteria*	2215	
	*Acidobacteria*	‘*Solibacteres’*	4191	41
	Acidobacteria	unclassified	1743	
	*Actinobacteria*	*Actinobacteria*	546521	382055
	*Aquificae*	*Aquificae*	2166	
	*Bacteroidetes*	*Bacteroidia*	38414	1306
	*Bacteroidetes*	*Cytophagia*	224023	
	*Bacteroidetes*	*Flavobacteriia*	178112	526723
	*Bacteroidetes*	*Sphingobacteriia*	57455	516940
	*Bacteroidetes*	unclassified	7043	6954
	*Chlamydiae*	‘*Chlamydiia’*	1621	
	*Chlorobi*	‘*Chlorobia’*	10540	
	*Chloroflexi*	*Anaerolineae*	441	679
	*Chloroflexi*	*Dehalococcoidetes*	836	
	*Chloroflexi*	*Chloroflexi*	5898	
	*Chloroflexi*	*Ktedonobacteria*	789	
	*Chloroflexi*	*Thermomicrobia*	1342	
	*Cyanobacteria*	*Gloeobacteria*	1075	
	*Cyanobacteria*	*Cyanobacteria*	25512	32848
	*Deferribacteres*	*Deferribacteres*	937	
	*Deinococcus-Thermus*	*Deinococci*	6560	1816
	*Dictyoglomi*	*Dictyoglomia*	513	
	*Firmicutes*	*Bacilli*	29929	8601
	*Firmicutes*	*Clostridia*	30400	19916
	*Firmicutes*	‘*Erysipelotrichi’*	196	
	*Firmicutes*	*Negativicutes*	2278	
	*Firmicutes*	unclassified		696
	*Fusobacteriia*	*Fusobacteriia*	2224	837
	*Gemmatimonadetes*	*Gemmatimonadetes*	1484	
	*Nitrospirae*	‘*Nitrospira’*	1799	1483
	*Planctomycetes*	*Planctomycetia*	12967	49
	*Proteobacteria*	*Alphaproteobacteria*	220054	151267
	*Proteobacteria*	*Betaproteobacteria*	1597220	1225776
	*Proteobacteria*	*Deltaproteobacteria*	36851	9357
	*Proteobacteria*	*Epsilonproteobacteria*	6449	3645
	*Proteobacteria*	*Gammaproteobacteria*	322470	667102
	*Proteobacteria*	‘*Zetaproteobacteria’*	632	
	*Proteobacteria*	unclassified	6963	12414
	*Spirochaetes*	*Brachyspirae*		385
	*Spirochaetes*	*Spirochaetia*	4917	815
	*Synergistetes*	*Synergistia*	1086	17795
	*Tenericutes*	*Mollicutes*	1478	1697
	*Thermotogae*	*Thermotogae*	2105	1004
	*Verrucomicrobia*	*Lentisphaerae*	1099	
	*Verrucomicrobia*	*‘Methylacidiphilae’*		722
	*Verrucomicrobia*	*Opitutae*	4479	46474
	*Verrucomicrobia*	‘*Spartobacteria’*	2286	678
	*Verrucomicrobia*	*Verrucomicrobiae*	5061	1135
	*Verrucomicrobia*	unclassified	350	
	unclassified	unclassified	6087	61031
Eukaryota			66903	
	Apicomplexa	Aconoidasida	885	
	Apicomplexa	Coccidia	443	
	Arthropoda	Arachnida	300	
	Arthropoda	Branchiopoda	606	
	Arthropoda	Insecta	4901	
	Ascomycota	Dothideomycetes	808	
	Ascomycota	Eurotiomycetes	3193	
	Ascomycota	Leotiomycetes	353	
	Ascomycota	Pezizomycetes	119	
	Ascomycota	Saccharomycetes	2579	
	Ascomycota	Schizosaccharomycetes	429	
	Ascomycota	Sordariomycetes	2809	
	Bacillariophyta	Bacillariophyceae	317	
	Basidiomycota	Agaricomycetes	920	
	Basidiomycota	Tremellomycetes	414	
	Basidiomycota	unclassified	156	
	Chlorophyta	Chlorophyceae	1145	
	Chlorophyta	Mamiellophyceae	1032	
	Chlorophyta	Trebouxiophyceae	299	
	Chordata	Actinopterygii	1986	
	Chordata	Amphibia	7102	
	Chordata	Ascidiacea	264	
	Chordata	Aves	473	
	Chordata	Mammalia	4801	
	Chordata	unclassified	487	
	Ciliophora	Oligohymenophorea	1052	
	Cnidaria	Anthozoa	1749	
	Cnidaria	Hydrozoa	8973	
	Echinodermata	Echinoidea	348	
	Hemichordata	Enteropneusta	209	
	Heterolobosea	Heterolobosea	440	
	Ichthyosporea	Ichthyosporea	291	
	Microsporidia	unclassified	127	
	Nematoda	Chromadorea	1450	
	Nematoda	Enoplea	111	
	Phaeophyceae	unclassified	347	
	Placozoa	unclassified	246	
	Platyhelminthes	Trematoda	191	
	Rhodophyta	Bangiophyceae	206	
	Streptophyta	Appendicularia	259	
	Streptophyta	Coniferopsida	253	
	Streptophyta	Isoetopsida	306	
	Streptophyta	Liliopsida	2100	
	Streptophyta	unclassified	7129	
	unclassified	unclassified	3558	
Viruses			7684	
Other**			3204	

* Only major phyla and classes are shown (those totaling ≥01 % of the relevant domain or phylum) ** Unassigned and/or unclassified

Our analysis detected groups of bacteria that in part matched what we expected based on an understanding of river ecology, and classifications to family conformed closely to the core groups detected in other freshwater aquatic systems [22–25]. The analysis also implicated additional industrially- and epidemiologically-relevant groups that likely are important in this reach of James River. More than half of the bacterial sequences detected by both the WGS and the 16S methods were *Proteobacteria* (*Betaproteobacteria*) and, within this group, the most abundant taxa were within the *Comamonadaceae*. In the WGS analysis, the most numerous genera in the *Comamonadaceae* were *Acidovorax* (iron oxidizers, nitrotolulene degraders, and plant pathogens that accounted for 10 % of WGS assigned bacterial reads and 0.8 % of 16S bacterial reads), *Polaromonas* (6 % of WGS bacterial reads and < 0.1 % of 16S bacterial reads). The *Polaromonas* were dominated by two groups capable of degrading polycyclic aromatic hydrocarbons, PAHs, previously detected in coal-tar-contaminated freshwater sediments [26]. Other prominent groups in this family identified by WGS were *Albidiferax*, which are iron reducers (3 % WGS bacterial reads and 0 % 16S), and *Variovorax*, which are biodegraders of diverse natural biogenic compounds as well as numerous anthropogenic contaminants (3 % WGS bacterial reads and 0.6 % of 16S bacteria). The 16S analysis identified additional genera in the *Comamonadaceae* including *Limnohabitans* (12 % of 16S bacterial reads), *Hydrogenophaga* (3 % of 16S bacterial reads), and *Rubrivivax* (1 % of 16S bacterial reads), each of which were seen at < 0.01 % of the WGS bacterial data. The next most abundant *Proteobacteria* group was the *Burkholderiaceae*, represented by *Polynucleobacter necessarius* (5 % of WGS bacterial reads and 2 % of 16S reads), an ubiquitous freshwater bacterioplankton and protozoan endosymbiont, and by *Burkholderia* (3 % of WGS bacterial reads and 0.1 % of 16S bacterial reads), a group that contains mammal and plant pathogens and bacterial strains that biodegrade polychlorinated biphenyls. Three additional prokaryote groups were represented by read counts in excess of 10 % in either the WGS or the 16S analysis: *Actinobacteria* (*Actinomycetales*, mostly *Streptomycetaceae*, *Nocardioidaceae*, *Micrococcaceae*, and *Mycobacterium*), *Gammaproteobacteria* (*Chromatiaceae*, *Enterobacteriaceae*, pathogenic *Pseudomonadaceae*, and *Vibrionaceae*), and *Bacteroidetes* (a number of agriculture-associated species within *Cytophagaceae*, ‘*Flexibacteraceae**’*, and *Flavobacterium*, some of which are common in freshwater lake sediments and other known commensals and opportunistic pathogens of fishes). *Alphaproteobacteria* (particularly nitrogen fixers) constituted 6 % of WGS bacteria and 3 % of the bacteria based on 16S analysis. Of the 229 OTUs identified in the 16S data set at the level of ≥0.01 % read abundance, 22 % were bacteria associated with domesticated plants and animals, agricultural soils, or had other agricultural relevance. Across the WGS and 16S analyses, the five most common groups observed in the James River metagenome (*Proteobacteria*, *Bacteroidetes*, *Actinobacteria*, *Cyanobacteria*, and *Verrucomicrobia*) accounted for 98 % of reads, and were among the most common groups observed in Mississippi River [22].

Bacterial groups that accounted for ≈ 1 % of assigned reads in either the WGS or the 16S data sets included *Deltaproteobacteria* (some of which have recently been identified as pathogens), *Curvibacter* (1 % of bacterial reads: a symbiont of *Hydra* which was the most abundant of all eukaryote reads), *Delftia* (non-fermentative, Gram-negative bacteria from soil, activated sludge, crude oil, oil brines, and water [27], and recently observed in association with the use of medically invasive devices such as endotracheal tubes [28] and intravascular-catheters [29]), *Comamonas* (a soil bacterium utilized to treat the industrial by-product 3-chloroaniline [30]; one strain has been observed to be the cause of bacteremic infections [31]), *Alicycliphilus* (degrades alicyclic and aromatic hydrocarbons), and *Verminephrobacter* (earthworm symbionts). Although they accounted for just under 1 % of 16S reads, a diverse suite of *Cyanobacteria* was represented, predominantly by *Synechococcus* species (39 % of cyanobacteria) and *Prochlorococcus* (8 % of cyanobacteria), both of which are ecologically significant autotrophic picoplankton, and roughly even proportions of reads were assigned to *Anabaena*, *Cyanothece*, *Nostoc*, and *Synechocystis* (each ~ 5–7 % of cyanobacteria). Approximately 2–3 % of cyanobacterial reads were assigned to *Acaryochloris*, *Cyanobium*, *Gloeobacter**,**Microcoleus**,**Microcystis*, and *Trichodesmium**.*

Eukaryotes accounted for 2 % of the reads with assigned taxonomy in the analysis of the James River WGS metagenome (Table 8). The core taxonomy of eukaryotes was nearly identical to those detected at two selected sites along the Mississippi River in Minnesota [22].  However, the proportion of reads attributed to eukaryotes in James River was considerably higher than the <0.1 % mean abundance of non-bacterial orders in the Mississippi; the increased eukaryote component in James River may be in part a consequence of the longer reads in the James River data set (133 bp vs. 100 bp). The James River WGS metagenome exhibited 155 eukaryote families, each represented in the data by between 5 and 1352 reads. Just over half of the families were common temperate aquatic flora, fauna, or fungi, and the remainder was assigned to terrestrial species (including those found in agricultural soils) and organisms that cause disease in fishes, humans, or agriculture. Considering those eukaryotic taxa with an abundance ≥1000 reads (83 % of eukaryote reads), we detected the following (in order of read abundance): freshwater polyp (17 % of reads), streptophytes (14 % of reads), amphibians (13 % of reads, mostly frog), insects (7 %, mostly culicids, dipterans, and lepidopterans), mammals (7 %, mostly human and mouse), fungi (13 %, several major classes including Saccharomycetes, Sordariomycetes, and Eurotiomycetes), teleost fishes (3 %), green algae (3 %), nematodes (2 %), and ciliate protozoans (2 %). Almost one-quarter of eukaryotic sequences were Chordata, predominantly amphibian (11 % of eukaryote reads), mammalian (7 % of eukaryote reads), and to a lesser extent fishes (3 %) and birds (0.7 %). Upstream land-based agricultural effects on James River were indicated by sequence matches to castor oil plant, beet, sorghum, rice, maize, bovine, equine, porcine, and galliform species. Additional taxonomic groups detected at a read cutoff of ≥100 included angiosperms, mosquitos, nematodes, and primates (human and New World monkeys). The signal for monkeys most likely derives from an exotic animal rearing and testing facility located in nearby Cumberland County. The facility raises grivet and macaque monkeys and holds a permit (as of May 2014) to discharge up to 76 × 10^3^ L day^−1^ of industrial pollution directly into James River 70 km upstream of Richmond (*op cit*. data request). Similarly, there are several aquaculture facilities with discharge permits (as of May 2014, *op cit*. data request) between Richmond and Lynchburg, and this could explain the high number of non-indigenous fish and fish disease hits.

As was observed for bacterial sequences, eukaryote sequences reflected the high level of anthropogenic use of and impact upon James River, and many eukaryote sequences were assigned to known disease agents or disease carriers relevant to humans, food crops, or fishes. The most abundant taxa with epidemiological relevance were Apicomplexa (2 % of WGS assigned eukaryote reads), Culicidae (2 % of WGS eukaryote reads), Onygenales (2 % of WGS eukaryote reads), Trypanosomatidae (1 % of WGS eukaryote reads), Hexamitidae (0.8 % of WGS eukaryote reads), Vahlkampfiidae (0.7 % of WGS eukaryote reads), Trichomonidae (0.5 % of WGS eukaryote reads), Sclerotiniaceae (0.5 % of WGS eukaryote reads), *Phytophthora* (0.5 % of WGS eukaryote reads), *Schistosoma* (0.3 % of WGS eukaryote reads), and *Trichinella* (0.2 % of WGS eukaryote reads).

*Archaea* and viruses/bacteriophages each accounted for ~0.2 % of assigned reads. Most *Archaea* reads were *Euryarchaeota* (81 %), represented by a diverse array of chemoautotrophs and *Crenarchaeota* (14 %). Virus and bacteriophage reads included assignments to Myoviridae, a type of Caudovirus, and Vibriophage, and were notable in their associations with other detected bacterial and eukaryotic taxa. In future studies that employ sampling methods to better capture viruses and phages, it may be possible to interpret the phage and virus records as proxies for bacterial or eukaryotic organisms with which there are known associations.

Comparative PCoA analysis of the James River WGS metagenome to 13 other aquatic WGS metagenomes accessible through MG-RAST indicated that James River was similar to Mississippi River samples [21, 25], and that these rivers were more similar to sludge [32] and aquaculture pond [33] metagenomes than to the metagenomes of lakes experiencing blooms [34] or Chesapeake Bay [35], the geographically proximal saline body of water into which James River empties. Cross-assembling the James River WGS metagenome with other freshwater aquatic metagenomes via crAss (an approach that allowed investigation of metagenomes not posted to MG-RAST) supported the interpretation that the James River metagenome was genetically most similar to Mississippi River (minimum genetic distance 0.11) and more similar to aquaculture [33] and sludge [32] metagenomes (minimum distance 0.26 and 0.63, respectively) than to the relatively more pristine waters of the upper Amazon River [17] (minimum distance 0.74) or to Lake Lanier [20] (minimum distance 0.75).

## Functional diversity

Genes associated with chromatin, cytoskeleton, nuclear structure, and cell motility (Table 9) were notably absent, a finding commensurate with the fact that the predominant taxa in the sample were bacteria. Compared to other representative aquatic metagenomes [22, 32–34], the James River functional assignments, like Mississippi River, were in line with intensive aquaculture, a lake experiencing algal bloom, and sludge, and very different from Chesapeake Bay. KEGG metabolic pathway maps provided deeper insight into the ecosystem functions conducted by the James River microbiota. Although the most complete identified pathways were associated with basic cellular maintenance (carbohydrate, amino acid, lipid, and energy metabolism), a substantial number of partial metabolic pathways were related to xenobiotic biodegradation and metabolism. Multiple reaction links were evident for pathways involved in processing or degrading atrazine, benzoate, bisphenol, chlorobenzene, chlorocyclohexane, ethylbenzene, PAHs, naphthalene, nitrotoluene, toluene, and xylene, many of which are PBTs. In many cases, the predicted xenobiotic pathways were indicated by abundances of identified enzymes exceeding 100 reads. For example, toluene degradation (enzyme entry 3.1.1.45) was implicated by 820 enzyme identifications, dioxin metabolism (enzyme entry 1.14.13.1) was implicated by 555 enzyme assignments, and benzoate degradation (enzyme entry 6.2.1.25) was implicated by 492 enzymes. Although the proportional representation among the SEED categories was not well matched between James and Mississippi Rivers, the functional links implicated in the James River metagenome, especially the abundance of xenobiotic biodegradation pathways, coincided well with the links exhibited in two Mississippi River metagenomes (St. Cloud and Twin Cities [22]). It is important to note that this is only a snapshot of the response of the microbial consortium to anthropogenic substances delivered to James River. It remains to be determined how quickly and to what degree the consortium shifts; if the river responds to increased amounts of synthetic compounds in much the same way as the human microbiome does [36], shifts in taxa and function could occur on the order of days. More complex sampling strategies across spatio-temporal scales are necessary to address this issue.

**Table 9:** Functional information: Composition of functional categories (COG)

COG Category	JREM1 Riffles_WGS
	(% of reads)
RNA processing and modification	0
Chromatin structure and dynamics	0
Energy production and conversion	10
Cell cycle control and mitosis	1
Amino acid metabolism and transport	11
Nucleotide metabolism and transport	4
Carbohydrate metabolism and transport	5
Coenzyme metabolism	4
Lipid metabolism	5
Translation	9
Transcription	4
Replication and repair	6
Cell wall/membrane/envelope biogenesis	6
Cell motility	0
Post-translational modification, protein turnover, chaperone functions	4
Inorganic ion transport and metabolism	4
Secondary structure	2
General functional prediction only	12
Function unknown	5
Signal transduction	3
Intracellular trafficking and secretion	2
Nuclear structure	0
Cytoskeleton	0
Defense mechanisms	2

## Additional results

This study revealed details of the function of the river as a medium for transmission of numerous infectious agents [37–39] , and garnered a wealth of epidemiologically-relevant data. Both prokaryotes and eukaryotes with health and disease implications were revealed by the taxonomic summaries. Numerous reads from both libraries matched plant and domestic animal pathogens: 21 % of the top 254 taxa in the WGS library and 28 % of the top 230 OTUs in the 16S library. Notable among the known human, food crop, and fish pathogens were *Agrobacterium* (0.5 % of WGS reads, 0.2 % of 16S reads), *Bacteroides* (0.9 % of WGS, 0.01 % of 16S), *Burkholderia* (1.5 % of WGS, 0.01 % of 16S) *Chromobacterium* (0.3 % of WGS, 0.2 % of 16S), *Comamonas* (1.7 % of WGS, 0.07 % of 16S), *Flavobacterium* (2 % of WGS, 0.4 % of 16S), *Legionella* (0.08 % of WGS, 0.01 % of 16S), *Mycobacterium* (1 % of WGS, 0.005 % of 16S), *Novosphingobium* (0.2 % of WGS, 0.004 % of 16S), pathogenic *Pseudomonas* species (3 % of WGS, 1 % of 16S), *Ralstonia* (0.7 % of WGS, 0 % of 16S), *Vibrio* (0.4 % of WGS, 0 % of 16S), and pathogenic *Enterobacteriaceae* (0.11 % of WGS, 0.11 % of 16S ). In addition, several of the cyanobacteria (*Nostocophycideae*, *Oscillatoriophycideae*, *Synechococcophycidea*) detected in this sample have been noted in other metagenomic studies of toxic blooms [38], and are considered potentially pathogenic because the toxins they produce under bloom conditions have adverse effects on both aquatic living resources and humans. Of the 50 top eukaryotes detected based on MEGAN read abundance in the WGS data, 6 were known human, plant, and animal parasites or pathogens; notably *Trichodina*, *Leishmania*, *Trypanosoma*, *Plasmodium*, *Naegleria*, and *Botryotinia*.

Out of the 2 % of reads revealed by MG-RAST to be associated with COG defense mechanisms (Table 9), 78 % were for multidrug resistance and 5 % were specifically for antibiotic resistance (Table 10), representing 13 different antibiotic resistance genes. These findings are consistent with the work of others [40] where antibiotic-resistant bacteria were isolated from freshwater samples from 16 US rivers at 22 sites, and studies showing high levels of antibiotic resistance in rivers in the UK [41], China [42], India [43], and Cuba [44]. The detection of antibiotic resistance genes is not necessarily surprising, given that so many natural organisms display resistance [45]; however, recent work in the Hudson River [46] documented a positive correction between counts of the fecal indicator *Enterococcus* and levels of resistant bacteria, and demonstrated a shared sewage-agricultural-domesticated animal associated source. Moreover, the study of Chinese rivers [42] detected a *synthetic* plasmid vector-originated ampicillin resistance gene in samples from six rivers, with higher levels being found in habitats that receive more untreated waste. This synthetic plasmid has a number of industrial and agricultural applications and there is a large chance of uncontrolled discharge into the environment. Alternately, antibiotic resistance may be transferred to other members of the river consortium by other genetic processes. Antibiotic resistance has been called one of the most pressing and urgent public health crises in the world [47], and our work, combined with the studies cited above, suggest that river water may serve as a significant reservoir or incubator for antibiotic resistance genes, where inputs of the waste from treated animals and humans could alter background levels of antibiotic resistance in the environment [44].

**Table 10:** Functions associated with antibiotic resistance

Database	JREM1 Riffles_WGS Functions Identified	Antibiotic Functions	Unique M5NR Accessions	Unique Antibiotic Resistance Genes
GenBank	2.4 × 10^6^	314	60	14
IMG	2.5 × 10^6^	302	56	21
KEGG	2.3 × 10^6^	306	51	14
PATRIC	2.3 × 10^6^	265	40	7
RefSeq	2.6 × 10^6^	338	64	23
SEED	1.8 × 10^6^	247	36	10
SwissProt	0.4 × 10^6^	2	1	1
TrEMBL	2.4 × 10^6^	322	61	20

The implications of finding such a diverse array of pathogenic species in recreational waters are profound and indicate the utility of a metagenomic approach for early detection and prevention of WBDOs. However, there are a number of caveats to consider regarding the current data set and analyses. First, these assignments do not necessarily imply that the predicted organisms were living because the analysis was based on DNA, not RNA, and the DNA could have come from dead cells and/or dormant organisms from a previous contamination event. Second, the assignments depend on the stringency of alignment settings and, because the genomes of disease-causing organisms are generally more thoroughly studied and reported than the genomes of free-living organisms, the prevalence of pathogen assignments may be biased due to the over-representation of pathogenic genomes in the databases. In other words, some of these assignments may be non-pathogenic organisms that have never been sequenced but are related to highly studied pathogens of humans, fishes, or crops. Finally, the sample is only a snapshot, as it was collected from a single segment of James River on a single day; conversely, one might speculate that some of the pathogenic groups detected that are not commonly observed in North America may be a signal of globalization and an indication of the changing demographic of Richmond’s human population.

In addition to the epidemiological ramifications of this metagenomic dataset, the novel ecological information it provides is notable. For example, other researchers who have studied the microbial consortia of rivers have concluded that river microbes generally are comparable to lake consortia [17, 48, 49]and we expected similar results.  In addition, we expected to observe a number of sequences that reflected a “Microbial Loop” [50] as illustrated for another aquatic system [51], dominated by heterotrophic bacteria and including representatives of cyanobacteria and algae, protozoans, zooplankton (especially nematodes and cladocerans), insects, and vertebrates. Indeed, both of these expectations were supported, and we observed that approximately half of the most abundant read assignments corresponded to microbes identified as ecologically significant in lakes [23], fit the expected microbial functional patterns [50], and corresponded to the major groups of freshwater microbes previously described and summarized [24] namely: ultramicrobacteria (made up of three groups *Polynucleobacter* and other *Betaproteobacteria*, acI *Actinobacteria*, and certain *Alphaproteobacteria*), opportunistic heterotrophs, phototrophs, and filamentous bacteria. Also as expected, the most commonly observed species in James River metagenome annotated reads was *Polynucleobacter*, corresponding to other large river biome reports [22, 25]. Likewise, a large proportion of the detected metabolic processes corresponded to the “natural” microbial loop. Interestingly, both taxonomic and functional analysis also revealed that a large component of the James River microbial consortium is processing a diverse suite of anthropogenic substances, providing especially a baseline reference for investigating the natural variability and function of bacteria that process polycyclic aromatic hydrocarbons, a group of microbes that are largely unexplored in the waters of this region. As was observed for the upper Mississippi River [22, 25], taxa represented in the James River metagenome were linked to the varied anthropogenic effects ranging from urban, suburban, and industrial, to forested land and agriculture (Table 11). It was striking that nearly half of the dominant bacterial groups (48 % of the top 50 species identified by WGS, 31 % of the major OTUs identified by 16S) were associated with degradation of pollutants and PBTs, sludge and other biological waste materials, or pathogenicity. At least 11 different prokaryote groups commonly associated with bioremediation were indicated as present in the top 50 groups; most numerous among these were degraders of dichloroethane, polyaromatic and chlorinated hydrocarbons, methyl tertiary butyl ether, and PCBs, represented by *Polaromonas**,**Acidovorax*, *Nocardioides*, and *Burkholderia*. Another seven species commonly used in industrial-scale production of metals, antibiotics, and spinosyns were indicated (including the genera *Delftia*, *Cupriavidus*, and *Saccharopolyspora*). It is notable that, although they accounted for fewer assignments, tens of thousands of hits implicated presence of bacteria known to process endocrine disruptors such as BPA (e.g., *Rhodococcus* [52] and *Sphingomonas* [53]). Such a diverse set of indicators of industrial effluent implies heavy impact upon this reach of James River by industrial and medical waste. However, as for the predicted pathogens, the present data set, being derived from WGS, does not provide a definitive determination of whether these microbes were active components of the James River ecosystem or whether they represent some transient populations introduced by runoff or other hydrological processes. The assemblage of industry- and medical-related microbes might be a consequence of the fact that the sample location is in the vicinity of CSOs, indicating that either the microbes or the substrates they metabolize are regularly disposed of to the sewer system. Similarly, the occurrence of so many different types of hydrocarbon degraders is likely a signal of railway and automotive non-point source runoff in addition to the permitted hydrocarbon and other point-source discharges. Whatever the sources, this metagenome snapshot indicates that a large portion of the ecological services provided by microbes of James River are related to biodegradation of anthropogenically introduced compounds.

**Table 11:** Putative roles of the most abundant bacterial and eukaryotic OTUs

	JREM1 Riffles_WGS		JREM1 Riffles_16S	
Putative Role	% OTUs	reads	% OTUs	reads
Bacteria				
Common free-living	52	1072474	68	2832637
Pollution degraders	22	484716	10	90537
Sludge, industrial, and medical waste	12	133200	9	184336
Pathogens (human, crops, and fish)	14	297920	12	132914
Eukaryote				
Common free-living	52	3468	–	–
Terrestrial/agriculture/aquaculture	33	1123	–	–
Pathogens (human, crops, and fish)	14	261	–	–

– not examined

## Conclusions

This first published whole-genome report of the iconic James River is among the few existing metagenome reports for large river biomes. Rivers provide numerous ecosystem services for humans and we are especially dependent on them for fresh water supply and sanitation purposes. This metagenome analysis illustrates that the core freshwater planktonic bacterio- and eukaryoplankton communities of this non-tidal portion of James River closely mirror the upper Mississippi River [22, 25], both of which differ from lake systems studied in a similar manner. This metagenome provides evidence that there exists a river consortium response to anthropogenic pollution and illustrates that the epidemiologically-relevant members of the James River microbial consortium are not a trivial component of the ecosystem and include organisms with genes for antibiotic resistance, which has recently been documented to be an important component of the human microbiome [54]. However, not all strains in the pathogenic genera detected are human or agricultural pathogens, and a limitation of this study is that pathogenic or virulent markers associated with the organisms found by sequencing were not further evaluated using PCR assays. Furthermore, because the current findings are based on limited sampling, generalizations cannot be made regarding spatio-temporal distributions of the indicated macro- and microbial communities. Deeper knowledge of associated interactions and potential ecological and environmental implications require more robust studies with intensive samplings throughout the watershed; such an approach will enhance our understanding of the occurrence, interactions, and ultimately the functions of these microbes, informing management and restoration efforts. The combined ecological and epidemiological analysis illustrates that a metagenomic approach is appropriate for addressing the challenges in identifying contamination sources and establishing cumulative risk metrics, and demonstrates the tremendous potential of ecogenomic approaches which, when applied over space and time, could be a valuable tool for epidemiology - specifically for monitoring the simultaneous presence, movement, and evolution of WBDO agents including bacteria, cyanobacteria, viruses, and eukaryotes. This and further studies should therefore allow health agencies to better identify organism-specific health risks and to enhance waterborne disease prevention efforts.

## Abbreviations

BPA: Bisphenol A
CSO: Combined sewer overflow
Db: Database
FNU: Formazin nephelometric unit, a nephelometric turbidity measure
M5NR: M5 non-redundant protein database
PBT: Persistent bioaccumulative toxins
PCB: Polychlorinated biphenyl
TMDL: Total maximum daily load
WBDO: waterborne disease outbreaks
